# Determinants of communication on sexual issues between adolescents and their parents in the Adaklu district of the Volta region, Ghana: a multinomial logistic regression analysis

**DOI:** 10.1186/s12978-022-01402-0

**Published:** 2022-04-27

**Authors:** Desmond Klu, Percival Agordoh, Charles Azagba, Evelyn Acquah, Phidelia Doegah, Anthony Ofosu, Evelyn Korkor Ansah, Margaret Gyapong

**Affiliations:** 1 Institute of Health Research, University of Health and Allied Sciences, Ho, Ghana; 2 School of Allied Health Sciences, University of Health and Allied Sciences, Ho, Ghana; 3 Adaklu District Health Directorate, Ghana Health Service, Accra, Ghana; 4 Ghana Health Service, Accra, Ghana

**Keywords:** Adolescents, Parents, Sex communication, Reproductive health, Adaklu district, Ghana

## Abstract

**Background:**

Poor communication on sexual issues between adolescents and their parents results in high rates of negative sexual practices such as teenage pregnancy. Contributing factors to this poor communication on sexual issues between adolescents and their parents in a high teenage pregnancy setting have not been adequately explored. We sought to fill this gap by examining the factors that predict communication on sexual issues between adolescents and their parents in the Adaklu district of the Volta region of Ghana.

**Methods:**

A baseline cross-sectional household survey of 221 adolescents aged 10–19 years in 30 randomly selected communities was used. A well-structured questionnaire was developed. A multinomial logistic regression analysis was used to examine factors that significantly influenced communication between adolescents and their parents regarding sex.

**Results:**

Only 11.3% of adolescents had discussions on sexual issues with both parents while 27.6% of communicated sexual issues with only one parent in Adaklu district. Adolescent males (AOR = 0.21, CI = 0.06–0.75), those aged 10–14 years (AOR = 0.41, CI = 0.04–0.57), non-members of adolescent health clubs (AOR = 0.46, CI = 0.21–1.00), and those living with only a father (AOR = 0.19, CI = 0.06–0.61) had lower odds of communicating with their parents on sexual issues.

**Conclusions:**

Adolescent-parental communication on sexual issues in Adaklu district is very low. This situation requires more empowerment of adolescents to enable them to discuss sexual issues with their parents through increased engagement with adolescent health club activities and capacity building capacity of parents with the right information on sexual and reproductive health by health staff within the district. Additionally, parents need to be equipped with communication skills to enable them to communicate effectively with their children.

**Supplementary Information:**

The online version contains supplementary material available at 10.1186/s12978-022-01402-0.

## Plain language summary

Parent-adolescent and older sibling adolescent communication on issues about sex is considered an important aspect of adolescent development and well-being, as it ensures informed decision making and good behavior and a protective factor for adolescent sexual health. However, poor communication on sexual issues between adolescents and their parents results in high rates of negative sexual practices such as teenage pregnancy. Contributing factors to this poor communication on sexual issues between adolescents and their parents in a high teenage pregnancy setting have not been adequately explored. This paper, therefore, examines the factors that predict communication on sexual issues between adolescents and their parents in the Adaklu district of the Volta region of Ghana. It highlights individual, household, societal and sexual-related factors that predict communication on sexual issues between adolescents and their parents in an area with a high rate of teenage pregnancy among adolescent girls.

The data on adolescent sexual and reproductive health in Adaklu district show poor communication on sexual issues between adolescents (aged 10–19 years) and their parents. Improving communication on sexual issues between adolescents and their parents in rural settings requires capacity building of parents (especially mothers) with the right information and communication skills for discussing sexual and reproductive health issues with their children.

**Supplementary Information:**

The online version contains supplementary material available at 10.1186/s12978-022-01402-0.

## Introduction

Adolescents aged 10–19 years are largely exposed to sexual and reproductive health risks due to the negative and risky sexual behaviours they often engage in [1] coupled with a high level of poor and misinformed knowledge on sexual and reproductive matters [2–4].

Parent-adolescent and older sibling adolescent communication on issues about sex is considered an important aspect of adolescent development and well-being, as it ensures informed decision making and good behavior and a protective factor for adolescent sexual health. It can result in the transmission of family values, curbing negative sexual health outcomes and promoting healthy sexual attitudes and behaviors among adolescents [1]. Studies have identified that family-oriented communication, especially from parents, plays a principal role in conveying knowledge and moral values to adolescents, including sexual and reproductive health (SRH) information [5, 6].

There is evidence that adolescents rely mostly on their colleagues and social media as the main source of information on sexual and reproductive health. Unfortunately, knowledge acquired from these sources is either inaccurate or inadequate and puts them at risk of negative sexual practices [5, 7, 8]. Parental communication on sexual issues with adolescents has been associated with better sexual health outcomes, low negative sexual practices and improved SRH [8–11]. Parental communication on sexual issues therefore empowers adolescents to manage the several challenges associated with sexual and reproductive health [12]. Studies have noted that while most adolescents preferred their parents as a reliable and main source of information on sex [13], hitherto, only a few obtained such information from them [14, 15].

In Ghana, parent-adolescent communication on sexual issues has not been adequately explored to the best of our knowledge. The literature has shown that a limited number of studies have been carried out on the subject, especially in settings where there are high rates of negative sexual outcomes such as teenage pregnancy, sexually transmitted infections, abortion and multiple sexual partnerships. These studies, however, were selective in assessment, scope and coverage [16–19]. For instance, Manu and colleagues [16] examined the kinds of SRH topics parents discussed with their children and the predictors of such SRH communication while Baku and colleagues [17] explored the opinions of parents in relation to discussions on adolescent sexuality.

This paper examines the factors that predict communication on sexual issues between adolescents and their parents in the Adaklu district of the Volta region of Ghana. It highlights individual, household, societal and sex-related factors that predict communication on sexual issues between adolescents and their parents in an area with a high rate of teenage pregnancy among adolescent girls. This data from this study is part of the dataset from a larger cross sectional study conducted as baseline for an adolescent reproductive health intervention project in the Adaklu district of the Volta Region of Ghana.

## Methods

### Study design and setup

The study was a baseline cross-sectional study of adolescents (10–19 years) and their parents/caregivers residing in Adaklu district of the Volta Region of Ghana. The Adaklu district comprises a total of 91 communities and the inhabitants are predominantly farmers. Farming forms approximately 78% of economic activities in the district and the inhabitants are mainly involved in crop production, bee keeping and livestock rearing. The district has a projected population of approximately 44,942, comprising of 22,022 males and 22,920 females, with females representing 51.0 percent of the population [20]. The estimated adolescent population in the district is 9, 887 and Women in Fertile Age account for 10, 394 out of the total population [20]. Adolescent health clubs are established in all the communities in the study area. These adolescent health clubs meet once every month in the various communities and health providers teach adolescents on personal hygiene, sexual and reproductive health, and environmental hygiene, courtesy for boys and girls. This is to ensure that adolescents live a chaste life and reduce risky sexual behaviours. The clubs were established in 2016 and currently there are 21 adolescent health clubs in the study area.

### Study population

The participants of this study were adolescents between the ages of 10–19 years, from 30 selected communities in the district who had lived in the area for a minimum of 6 months.

### Sample size and sampling technique

A total of 30 communities (clusters) with a minimum population of 500 people were randomly selected from the district. The modified Expanded Program on Immunisation (EPI) cluster sampling technique was used to select seven households in each community. To which questionnaires were administered to adolescents (10–19 years) in each household. A conscious effort was made to include equal numbers of male and female respondents. Similarly, both adolescents who were members of adolescent health clubs and those who were not members had an equal chance of being recruited for the baseline study. The sample size was calculated using the single population proportion formulation under the following axioms: 17.6% proportion; confidence level was taken to be 95% with α = 0.05 value, 3% margin of error and design effect of 2. Five percent was added for the expected non-response rate, constituting the final sample size of 221 adolescents aged 10–19.

### Data collection tools and procedure

Data were collected electronically using RedCap software. The questionnaire was adapted from adolescent survey tools used in previous adolescent sexual and reproductive health studies of international standards and several studies were reviewed to achieve the study objectives [21]. The tools were pre-tested and all the necessary corrections and changes were made.

The tool designed in English but administered in the Ewe language was used to collect information on socio-demographic characteristics, sexual and reproductive health knowledge and services, sexuality, communication on sexual issues between adolescents and their parents, risky sexual behaviour, sexual harassment and coercion (see Additional file 1). Data were collected by 12 well trained research assistants selected based on their proficiency in English and Ewe language which are commonly spoken in the study area.

### Outcome and predictor variables

The outcome variables of this study were communication on sexual issues with parents, comprising three categories; communication on sexual issues with (1) both parents, (2) only one parent and (3) no parents, similar to the measurement used in previous studies by Manu and colleagues which explored the extent and patterns of parent–child communication on sexual issues [16].

Predictor variables included sex [male or female], age [10–14 and 15–19], level of education,[ no education, primary and secondary/higher] current school attendance [currently in school or out of school], adolescent club membership, household living arrangement [living with both parents, only father, only mother, only brother(s), only sister(s) or not], frequency and ease of communication with parents, sexual history [never had sex and ever had sex], history of contraceptive use [never used contraceptives and ever used contraceptives], sexual harassment experiences [kissed, breast fondled, touched) or not. The respondents were also asked about their participation in adolescent health clubs which are common in the study district. The measurement of the predictor variables was guided by previous studies [33–40]

### Data processing and analysis

Both descriptive (frequency distribution tables) and inferential statistics (bivariate and multivariate analysis) were used in the analysis. The bivariate analysis examined the association between the outcome and predictor variables. Multinomial logistic regression analysis was then used to examine which factors significantly influenced communication on sexual issues between adolescents and their parents. All the variables in the multinomial logistic regression model were entered in one step. Our reference category in the regression analysis was communication on sexual issues with no parents. SPSS was used to run all analyses, and the results of the multinomial logistic regression analysis are presented as odds ratios.

### Ethical approval to conduct this study

This study received ethical clearance from the Research Ethics Committee (REC) of the University of Health and Allied Sciences (UHAS), Ghana. The study was approved with the reference number ‘*UHAS-REC A.8 [3] 18–19’.*

## Results

### Socio-demographic characteristics of study participants

The background characteristics of adolescents are presented in Table 1. Of the 221 adolescents, 59.7% were females and 40.3% were males. A higher proportion of adolescents (54.8%) were within the 15–19 years age bracket, and 43.4% had completed their primary level of education. Majority (90%) of adolescents are currently in school and approximately 65% are members of adolescent health clubs. Out of the 221 respondents, 92 (41.6%) live with their mothers, 43 (19.5%) live with their fathers, and 86 (38.9%) live with both parents. Overall, 44% of adolescents find it easy to communicate with their fathers while 65.2% easily communicate with their mothers, probably because most adolescents live with their mothers relative to their fathers. Seven out of ten adolescents had never had sexual intercourse, whereas 22.2% had had sexual intercourse. With regard to contraceptive use, majority (81.0%) of adolescents had never used contraceptives relative to 19% who had used contraceptives. Approximately 20.4% of all adolescents had been sexually harassed in the past.

**Table 1:** Background characteristics of adolescents

Characteristics	Number	Percent
Sex		
Male	89	40.3
Female	132	59.7
Age group		
10–14	100	45.2
15–19	121	54.8
Educational level attained		
No education	32	14.5
Primary	96	43.4
Secondary	93	42.1
Current school attendance		
Schooling	199	90.0
Not schooling	22	10.0
Adolescent club membership		
Not a member	77	34.8
Member	144	65.2
Living arrangements		
Living with both Parents	86	38.9
Living with father only	43	19.5
Living with mother only	92	41.6
Living with older brother(s)	54	24.4
Living with older sister(s)	50	22.6
Frequency of communicating with father		
Easy	97	43.9
Average	32	14.5
Difficult	92	41.6
Frequency of communicating with mother		
Easy	144	65.2
Average	29	13.1
Difficult	48	21.7
Sexual history		
Never had sex	172	77.8
Ever had sex	49	22.2
History of contraception		
Never use contraceptives	179	81.0
Ever use contraceptives	42	19.0
Sexual harassment		
Not sexually harassed	176	79.6
Sexually harassed	45	20.4
Communication on sexual issues with parents		
Both parents	25	11.3
Only one parent	61	27.6
No parent	135	61.1

Approximately 61% of adolescents admitted to not communicating with any of their parents on sexual issues, 27.6% said they discussed sexual issues with only one parent, and only 11.3% indicated that they communicated with both parents.

### Association between adolescent characteristics and communication on sexual issues with their parents

Table 2 presents the association between the characteristics of adolescents aged 10–19 and communication on sexual issues with their parents. There was a positive correlation between adolescent communication with parents and the sex of adolescents, adolescent club membership, living arrangement, frequency of communication with either father and mother and history of contraceptive use (p < 0.05). A higher proportion (14.4%) of female adolescents had communication on sexual issues with both parents as compared with 6.7% of their male counterparts who did so.

**Table 2:** Association between adolescent characteristics and communication on sexual with their parents

Factors	Adolescent communication on sexual issues						
	With both Parents		With Only One Parent		With No Parents		p-value
	N	%	N	%	N	%	
Sex							
Male	6	6.7	15	16.9	68	76.4	0.001**
Female	19	14.4	46	34.8	67	50.8	
Age group							
10–14	7	7.0	30	30.0	63	63.0	0.174
15–19	18	14.9	31	25.6	72	59.5	
Educational level attained							
No education	4	12.5	10	31.3	18	56.3	0.977
Primary	10	10.4	26	27.1	60	62.5	
Secondary	11	11.8	25	26.9	57	61.3	
Current school attendance							
Schooling	22	11.1	55	27.6	122	61.3	0.935
Not schooling	3	13.6	6	27.3	13	59.1	
Adolescent club membership							
Not a member	7	9.1	14	18.2	56	72.7	0.031*
Member	18	12.5	47	32.6	79	54.9	
Living arrangements							
Living with both Parents	15	17.4	16	30.2	45	52.3	0.041*
Living with father only	3	7.0	7	16.3	33	76.7	
Living with mother only	7	7.6	28	30.4	57	62.0	
Living with older brother(s)	10	18.5	14	25.9	30	55.6	0.156
Living with older sister(s)	6	12.0	13	26.0	31	62.0	0.953
Frequency of communicating with father							
Easy	17	17.5	22	22.7	58	59.8	0.023*
Average	5	15.6	8	25.0	19	59.4	
Difficult	3	3.3	31	33.7	58	63.0	
Frequency of communicating with mother							
Easy	22	15.3	43	29.9	79	54.9	0.023*
Average	2	6.9	9	31.0	18	62.1	
Difficult	1	2.1	9	18.8	38	79.2	
Sexual history							
Never had sex	17	9.9	43	25.0	112	65.1	0.068
Ever had sex	8	16.3	18	36.7	23	46.9	
History of contraception							
Never use contraceptives	17	9.5	45	25.1	117	65.4	0.022*
Ever use contraceptives	8	19.0	16	38.1	18	42.9	
Sexual harassment							
Not sexually harassed	22	12.5	48	27.3	106	60.2	0.544
Sexually harassed	3	6.7	13	28.9	29	64.4	

**p = 0.001; * p < 0.05

More adolescents who are members of Adolescent health clubs in the district communicate with their parents on sexual issues compared to adolescents who are not members of the clubs. Furthermore, adolescents who lived with both parents had better communication (17.4%) with their parents concerning sexual issues compared to the adolescents who lived with either their mother (7.6%) or their father (7%). However, a higher percentage (30.4%) of adolescents living with their mothers indicated having conversations with only one of their parents on sexual issues. Adolescents who easily communicate with both parents on non-sexual issues also easily communicate with them on sexual issues. With regard to their contraceptive history, a higher proportion of adolescents who had ever used contraceptives had communicated with both parents concerning sexual issues relative to those who had never used contraceptives.

### Predictors of communication on sexual issues between adolescents and their parents/caregivers

Table 3 shows the results of the multinomial logistic regression analysis. The model explained 35% of the changes in communication on sexual issues between adolescents and their parents, suggesting that it is a good fit with the data. Gender was a significant determinant of communication on sexual issues between adolescents and both parents as well as adolescents and only one of their parents. Compared to adolescent females, adolescent males are less likely to talk to both parents as well as one of their parents.

**Table 3:** Odds ratios and confidence intervals for factors affecting communication on sexual issues between adolescent and their parents (No parents, Only one parent, and Both parents): Results from a multinomial logistic regression model

Factors	Adolescent communication on sexual issues			
	With both parents		With only one parent	
	Exp β	95% CI	Exp β	95 CI
Sex				
Male	0.21**	0.06–0.75	0.37***	0.16–0.72
Female (RC)	0.00		0.00	
Age group				
10–14	0.14***	0.04–0.57	1.04	0.47–2.33
15–19 (RC)	0.00		0.00	
Educational level attained				
No education	4.29	0.71–25.84	1.08	0.35–3.32
Primary	0.97	0.28–3.32	0.84	0.37–1.89
Secondary (RC)	0.00		0.00	
Current school attendance				
Schooling	1.48	0.24–9.17	1.22	0.36–4.07
Not Schooling (RC)	0.00		0.00	0.00
Adolescent club membership				
Not a member	0.84	0.25–2.82	0.46**	0.21–1.00
Member (RC)	0.00		0.00	
Living arrangements				
Living with both Parents (RC)	0.00		0.00	
Living with father only	0.37	0.07–2.11	0.19***	0.06–0.61
Living with mother only	0.27**	0.08–0.96	0.57	0.27–1.21
Living with older brother(s)				
No	0.38	0.11–1.30	1.30	0.55–3.08
Yes (RC)	0.00		0.00	
Living with older sister(s)				
No	1.60	0.41–6.24	1.21	0.52–2.85
Yes (RC)	0.00		0.00	
Frequency of communicating with father				
Easy	7.58**	1.53–37.63	0.53	0.25–1.16
Average	4.70	0.75–29.34	0.61	0.22–1.71
Difficult (RC)	0.00		0.00	
Frequency of communicating with mother				
Easy	11.71**	1.23–111.80	1.84	0.73–4.65
Average	13.56	0.84–217.10	2.16	0.64–7.34
Difficult (RC)	0.00		0.00	
Sexual history				
Never had sex	4.80***	9.68–20.18	0.46	0.06–3.37
Ever had sex (RC)	0.00		0.00	
History of contraception				
Never use contraceptives	3.87	8.76–35.58	0.53	0.07–4.08
Ever use contraceptives (RC)	0.00		0.00	
Sexual harassment				
Not sexually harassed	7.31**	1.41–37.71	2.33	0.87–6.27
Sexually harassed (RC)	0.00		0.00	

RC = Reference Category; ***p value = 0.000; **p value = 0.001; *p value < 0.05

The results indicate that adolescents aged 10–14 have lower odds (86% less) of engaging in sexual discussions with both parents than adolescents aged 15–19. Age, however, did not significantly differentiate adolescents who had communicated with only one parent on sexual issues and those who had not communicated with their parents on sexual issues. Furthermore, the relevance of adolescent health club membership is evident in it being a significant predictor of adolescent-parent communication on sexual issues. Compared to adolescents who are members of health clubs, those who are not health club members are 54% less likely to engage only one parent in sexual conversation. Interestingly, there was no statistically significant relationship between adolescent club membership and adolescent communication on sexual issues with both parents.

The household living arrangement of adolescents negatively predicted communication on sexual issues between adolescents and their parents. Compared to adolescents living with both parents, those living with only their fathers and only their mothers were 81% and 73% less likely to communicate with both parents and only one parent on sexual issues respectively. There is a positive statistical relationship between the frequency of adolescent-parent communication (non-sexual issues) and adolescent-parent communication on sexual issues. Adolescents who easily communicated with their fathers were 7.58 times more likely to have communication on sexual issues with both parents compared to adolescents who found it difficult to communicate with their parents on non-sexual related issues. Similarly, adolescents who easily communicate with their mothers were found to be 11.71 times more likely to discuss sexual issues with both parents compared to those who find it difficult to communicate with their mothers.

Adolescent sexual history and experience of sexual harassment both positively predicted communication on sexual issues between adolescents and their parents. Adolescents who never had sexual intercourse were approximately five times more likely to discuss sexual issues with both parents relative to adolescents who had ever had sexual intercourse. Similarly, adolescents who indicated that they had never been sexually harassed had a higher likelihood (more than six times) of communicating about sex with both parents compared to those who had experienced sexual harassment.

## Discussion

The study examined the factors affecting poor communication on sexual issues between adolescents and their parents in the Adaklu district of the Volta Region. It was clear from our findings that majority of adolescents (10–19 years) do not communicate with their parents/caregivers with regard to sexual issues. This finding concurs with previous studies [7, 17, 22–24], where cultural and religious norms prohibit adolescent-parent communication on sexual issues. These studies found that in rural areas, where cultural norms are deeply rooted in the belief system of people, sexuality is considered sacred and not to be discussed with adolescents. It is culturally believed that discussing sexual issues with children and adolescents leads them to engage in early sex and risky sexual behaviour that can result in teenage pregnancy and STDs [25]. Furthermore, the socio-cultural orientation given to adolescents, especially girls, makes them too timid to ask their parents questions bordering on sexuality [26]. However, within the Ghanaian context, there is evidence of high adolescent-parent communication on sexual issues. Kumi-Kyereme et al. [27], using nationally representative data from Ghana found that most adolescents communicated with their parents and other family members on sexual matters.

Adolescent females compared to males were more likely to discuss sexual matters with both parents and only one parent (mother or father) regardless of who they live with. This finding supports other studies where parents give more priority to their daughters with regard to educating them about sexual issues [16, 24, 28]. Parents consider their daughters to be more susceptible to risky sexual behaviours and sexual health issues including sexual harassment, coercion, teenage pregnancy, and abortion. This understanding makes parents very protective of their daughters and as such gives them the needed audience when they are approached by them to discuss sexual issues. Sons are less available or open to such discussion with their sons [8, 27–29]. The poor communication on sexual issues between adolescent males and their parents relative to females found in this study also exposes adolescent boys to risky sexual behaviours including early sexual debut, multiple sexual partnerships, risk of sexually transmitted infections and perpetration of violent sexual acts. Studies have found that due to the adventurous and curious nature of boys, they are exposed to sexually explicit content on television and the internet which they blindly put into practice [30, 31].

The formation and existence of adolescent health clubs in rural communities increases the knowledge and understanding of adolescents on SRH issues, including contraception, teenage pregnancy, abortion, risky sexual behaviour and promotion of behavioural change among adolescents [23, 32, 33]. Findings from our study indicate that adolescents who are not members of health clubs in the community are less likely to discuss sexual issues with their parent. In other words, belonging to a health club increases an adolescents’ likelihood of communicating with their parents regarding sexual matters [32].

Family living arrangements and household structure are both known to have significant influences on communication between adolescents and their parents [34–36]. Our study finding shows that adolescents living with only a mother or father have a lower probability of discussing sexual matters with their parents compared to those living with both parents. Living with both parents ensures strict and effective monitoring, supervision and interaction between adolescents and their parents, which may subsequently influence adolescents’ sexual behaviours [35].

Good communication and relationships between adolescents and their parents on non-sexual matters increases the chance of frequent discussion on sexual issues amongst them [16, 37]. Our findings clearly showed that adolescents who easily communicate with their mothers and fathers are more likely to engage their parents in sexual conversations compared to those who find it difficult to talk to their parents. The findings of previous studies conducted in Ghana and the United States of America [16, 38] corroborate our research findings.

Sexual abstinence among adolescents is highly associated with good communication on sexual issues between adolescents and their parents [39, 40]. In our study, adolescents who had never had sexual intercourse had higher odds of discussing sexual matters with their parents relative to adolescents who had ever had sex. Communication on sexual issues between adolescents and their parents results in adolescents being well-informed and knowledgeable about the risks and negative consequences of early sexual initiation to make better sexual decisions. This finding is similar to the results of previous studies [39, 41]. Parents usually have intolerant attitudes towards premarital sexual intercourse. Thus, communication on sexual issues with parents provides adolescents with the needed information that encourages sexual abstinence.

Finally, our study also found that adolescents who had never experienced sexual harassment were more likely to have discussed issues related to sex with their parents relative to those who were victims of sexual harassment. Studies have revealed that during adolescent-parental communication on sexual issues, the scope of topics parents discuss with their children is often limited to issues related to protection from sexual abuse and harassment and pubertal development [42, 43]. Parents’ genuine desire to prepare and protect their children from sexual abuse and to protect the family honour motivates them to discuss sexual issues with them [43].

## Conclusion

The findings from our study indicate that adolescent-parent communication on sexual issues in Adaklu district is low, and when it occurs, is more common among female adolescents and only one parent (mostly mothers). Male adolescents, adolescents aged 10–14 years, not being a member of adolescents’ health club and living with only one parent were predictors of low communication on sexual issues between adolescents and their parents. However, it is worth noting that, some level of communication on sexual issues takes place between parents and female adolescents, adolescents aged 15–19 years, adolescents who are members of health clubs, adolescents who easily have general communication with their fathers and mothers, adolescents who practice sexual abstinence and adolescents and who have never been sexually harassed. This implies that, to increase the frequency of adolescent-parent communication on sexual issues in rural settings, adolescent males should be encouraged and empowered to talk to their parents on sexual matters often. Adolescent health clubs must be made more attractive to non-members to encourage them to attend regular club meetings and gain knowledge and understanding of SRH issues that will minimise the risk of early sexual debut, teenage pregnancy, multiple sexual partners, STDs, and HIV/AIDS. Other intervention programmes to improve communication on sexual issues between adolescents and their parents should include strategies to build the capacity of parents (especially mothers) with the right information, communicative skills and confidence in discussing sex and reproductive health issues with their children to ensure positive sexual and reproductive health behaviours among adolescents.

## Supplementary Information

**Additional file 1.** Supplementary file.

## Data Availability

The data and all materials analysed/used for this study will be made available and accessed upon request to the corresponding author.

## Abbreviations

ASRHR: Adolescent Sexual Reproductive Health and Rights
EPI: Expanded program on immunisation
HIV/AIDS: Human immunodeficiency virus/acquired immunodeficiency syndrome
REC: Research Ethics Committee
SRH: Sexual and reproductive health
STDs: Sexually transmitted diseases
UHAS: University of Health and Allied Sciences
