# Supplementary material for: Determinants of communication on sexual issues between adolescents and their parents in the Adaklu district of the Volta region, Ghana: a multinomial logistic regression analysis
Source: Reprod Health. 2022 Apr 27;19:101. doi: 10.1186/s12978-022-01402-0 (PMC9044737; doi:10.1186/s12978-022-01402-0)
Supplement: Supplementary file 1 — Additional file 1. Supplementary file. [file 12978_2022_1402_MOESM1_ESM.docx]

**Supplementary file**

**UNIVERSITY OF HEALTH AND ALLIED SCIENCES, HO - GHANA**

INSTITUTE OF HEALTH RESEARCH

PROJECT TITLE:

“Strengthening the capacity of Health Management Teams to use DHIMs data in engaging stakeholders for effective decision-making in addressing teenage pregnancy in the Volta region of Ghana.”

**COMMUNITY SURVEY**

**[ADOLESCENT (10 – 19 YEARS) QUESTIONNAIRE]**


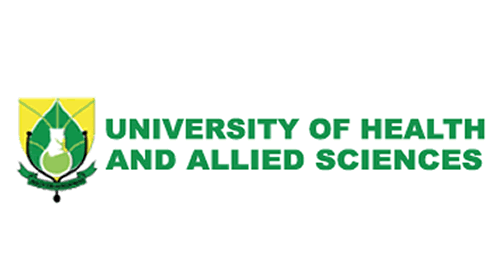

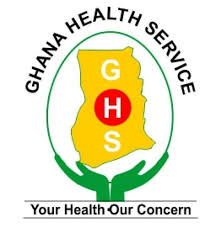


| **INTRODUCTION** |
| --- |

Good morning/afternoon, my name is ______________ and I represent the Institute of Health Research at University of Health and Allied Sciences, Ho.

We are conducting a survey within (*name of geographic area*). The purpose of this survey is to learn what young people understand about reproduction, contraception, sexually transmitted infections, HIV and AIDS. This information will help us understand the health needs of adolescents in (*name of geographic area*).

Regarding this, I would like to ask you some questions. Some of the questions are personal, but the answers you give will not be shown to anyone. They will only assist us in learning more about the behavior, beliefs and practices of adolescents. We especially want your answers because if everyone who is selected participates, our information will be more useful.

Participation in this survey is voluntary and you can choose not to answer any individual question or all the questions. However, we hope that you will participate in this survey since your views are important.

The survey usually takes _______ minutes to complete. Whatever information you provide will be kept strictly confidential and will not be shown to other persons.

Do I have your permission to continue? (Circle one.)

1 = Yes 2 = No (End the interview)

(Interviewer arranges for a private setting to conduct interview)

**START TIME FOR INTERVIEW HOURS MINS**

| **Section 1: Sociodemographic and family characteristics** |
| --- |

| 1.1 SEX OF RESPONDENT | MALE 1  FEMALE 2 |  |
| --- | --- | --- |
| - 1. How old were you at your last birthday? | Years old |  |
| - 1. What is your religion? | None 01  Christian 02  Muslim 03  Traditionalist 04  Atheist 05  Prefer not to say 06  Other (specify) …………………… 97 |  |
| - 1. What is the highest level of schooling you completed?   (CIRCLE HIGHEST SCHOOL LEVEL) | None 00  Primary 01  JHS 02  SHS 03  Technical 04  Commercial or Secretarial 05  Other (specify)……………. 97 |  |
| - 1. Are you currently attending regular school? | Yes, full-time 1  No 2 |  |
| ***Now I have some questions about work*** |  |  |
| - 1. Are you looking for work? | Yes 1  No 2 |  |
| - 1. Have you ever worked for pay? | Yes 1  No 2 | If 2, skip to 1.13 |
| - 1. How old were you when you started working for pay? | Age in years |  |
| - 1. Are you currently working for pay? | Yes 1  No 2 | If 2, skip to 1.13 |
| - 1. About how many hours a week do you work? | Hours |  |
| - 1. What type of work do (did) you do? | Farmer 01  Fisherman 02  Trader 03  Artisan 04  Apprentice 05  Other, specify ……………………….. 97  Not applicable ……………………….. 99 |  |
| - 1. How much do (did) you earn in a week? (*estimate*) | Weekly wage Ghana cedis |  |
| **Now I have some questions about your family.** | | |
| - 1. Is your father alive? | Yes 1  No 2 | If 2, skip to 1.17 |
| - 1. Does he live in the same household as you? | Yes 1  No 2 |  |
| - 1. How easy is it for you to talk with your father about things that are important to you? | Very easy 1  Easy 2  Average 3  Difficult 4  Very difficult 5  Do not see him 6 | If 6, skip to 1.17 |
| - 1. How often do you discuss sex-related matters with your father? | Often 1  Occasionally 2  Never 3 |  |
| - 1. Is your mother alive? | Yes 1  No 2 | If 2, skip to 1.21 |
| - 1. Does she live in the same household as you? | Yes 1  No 2 |  |
| - 1. How easy is it for you to talk with your mother about things that are important to you? | Very easy 1  Easy 2  Average 3  Difficult 4  Very difficult 5  Do not see her 6 |  |
| - 1. How often do you discuss sex-related matters with your mother? | Often 1  Occasionally 2  Never 3 |  |
| - 1. Do you have any older brothers? | Yes 1  No 2 | If 2, skip to 1.25 |
| - 1. Do any live in the same household? | Yes 1  No 2 |  |
| - 1. How often do you discuss sex-related matters with your older brother(s)? | Often 1  Occasionally 2  Never 3 |  |
| - 1. Do you have any older sisters? | Yes 1  No 2 | If 2, skip to 1.27 |
| - 1. Do any live in the same household? | Yes 1  No 2 |  |
| - 1. How often do you discuss sex-related matters with your older sisters(s)? | Often 1  Occasionally 2  Never 3 |  |
| **And now I have some questions about your social activities.** | | |
| - 1. Do you ever go to clubs, movies or parties where only young people gather? | Yes 1  No 2 |  |
| - 1. How often do you go to clubs, movies or parties where young people gather? | Often 1  Occasionally 2  Never 3 |  |
| - 1. Do you know about the existence of an adolescent club/group in this community? | Yes 1  No 2 |  |
| - 1. Are you a member of an adolescent club/group in this community/district? | Yes 1  No 2 |  |

| **Section 2: Sources of information on, and knowledge of reproductive health** |
| --- |

**Now in this next set of questions, I shall be asking you questions about where you get your information and knowledge of reproductive health. Please, kindly give me your honest responses. Remember, there is no wrong answer and no one else will learn about our conversation.**

| - 1. Young people learn about **puberty** - I mean the ways in which boys' and girls' bodies change during the teenage years - from many sources. They may learn from teachers at school, parents, brothers and sisters, from friends, from health workers or they may learn from books, films and magazines. What has been the most important source of information for you on this topic? And the second most important? CIRCLE MOST IMPORTANT IN COL 1 AND SECOND MOST IMPORTANT IN COL 2   2. From whom, or where, would you prefer to have received more information on this topic?   CIRCLE ONE ANSWER IN COL. 3 | School teacher  Mother  Father  Brother  Sister  Other family members  Friends  Health workers  Books/magazines  Films/Videos  Other (Specify………) | | (1)  Most Important  01  02  03  04  05  06  07  08  09  10  11  ………... | | (2)  Second most important  01  02  03  04  05  06  07  08  09  10  11  ………... | (3)  Preferred  01  02  03  04  05  06  07  08  09  10  11  ………... |
| --- | --- | --- | --- | --- | --- | --- |
| - 1. Now I want to ask you a similar question about sources of information on the **sexual and reproductive systems of men and women** - I mean where eggs and sperm are made and how pregnancy occurs. What has been the most important source of information on this topic? And the second most important? CIRCLE IN COLS. 1 AND 2.   2. From whom or where, would you prefer to receive (or prefer to have received) more information on this topic?   CIRCLE ONE ANSWER IN COL. 3 | School teacher  Mother  Father  Brother  Sister  Other family members  Friends  Health workers  Books/magazines  Films/Videos  Other (Specify………) | | (1)  Most Important  01  02  03  04  05  06  07  08  09  10  11  ……….. | | (2)  Second most important  01  02  03  04  05  06  07  08  09  10  11  ……….. | (3)  Preferred    01  02  03  04  05  06  07  08  09  10  11  ………… |
| - 1. Now there is a third similar question about sources of information on **relationships** - I mean how boys should treat girls and vice versa. What has been the most important source of information on this topic? And the second most important? CIRCLE IN COLS 1 AND 2   2. From whom, or where, would you prefer to receive more information on this topic?   CIRCLE ONE ANSWER IN COL. 3 | School teacher  Mother  Father  Brother  Sister  Other family members  Friends  Health workers  Books/magazines  Films/Videos  Other  (Specify………) | | (1)  Most Important  01  02  03  04  05  06  07  08  09  10  11  ………... | | (2)  Second most important  01  02  03  04  05  06  07  08  09  10  11  ……….. | (3)  Preferred  01  02  03  04  05  06  07  08  09  10  11  ……….. |
| 2.7 Some schools have classes on puberty, on sexual and reproductive systems and on relationships between boys and girls. Did you ever attend school classes on any of these topics? | Yes 1  No 2  Not sure 3  Never been to school 4 | | | | | If 4, skip to 2.9 |
| 2.8 Do you think that there should be (more) classes on these topics, fewer classes or were the number about right? | More 1  Less 2  About right 3 | | | | |  |
| 2.9 Now I have some other questions on sex and reproduction. I will read you some statements. Please tell me whether you think the statement is true, or false, or whether you don't know. | True | False | | Don't Know/ Not Sure | |  |
| A woman can get pregnant on the very first time that she has sexual intercourse. | 1 | 2 | | 3 | |  |
| 2.10 A woman stops growing after she has had sexual intercourse for the first time. | 1 | 2 | | 3 | |  |
| 2.11 A woman is most likely to get pregnant if she has sexual intercourse half way between her periods. | 1 | 2 | | 3 | |  |

| **Section 3** **Current/most recent heterosexual relationship** |
| --- |

| 3.1 Have you ever had a girl/ boyfriend? By girl/boyfriend, I mean someone to whom you were emotionally attracted and whom you 'dated' (*use local terms to specify going out together unaccompanied by other adults*) | Yes 1  No 2 | If 2, skip to 3.13 |
| --- | --- | --- |
| 3.2 How many girl / boy friends have you had? | Number |  |
| *Ask the following sequence of questions about CURRENT (MOST RECENT) girl / boy friend* | | |
| 3.3 How old is your current/last partner? *Probe for current age* | Age (in years)  Don’t Know……………………………..98 |  |
| 3.4 What is the marital status of the person you are currently dating? | Single 01  Married 02  Divorce 03  Separated 04  Don’t know 98 |  |
| 3.5 What work does/did your boyfriend/girlfriend do? | Not working 01  Driver/Motor rider (Okada) 02  Farmer 03  Trader 04  Seamstress 05  Hairdresser 06  Apprentice 07 Other, specify …………………… 97 |  |
| 3.6 How long have you been 'dating'/dated your current/last partner? | Months ago |  |
| 3.7 Are you still in a relationship with this partner? | Yes 1  No 2 | If 1, skip to 3.10 |
| 3.8 How long did the last relationship last? I mean from the first time you 'dated' to the last time? | Days 1  Weeks 2 Months 3  Years 4 |  |
| 3.9 During the time you were/have been 'dating' did you 'date'/have you ‘dated’ anyone else? | Yes 1  No 2 |  |
| 3.10 How would you describe your relationship? | (a) Casual 1   1. Serious 2 2. Important/might lead to marriage 3 3. Engaged to be married 4 | If 4, skip to 3.12 |
| 3.11 And how do you think your partner would describe her /his relationship to you? | (a) Casual 1  (b) Serious 2  (c) Important/might lead to marriage 3 |  |
| 3.12 Did you and your partner have any physical contact, such as holding hands, hugging or kissing? | Yes 1  No 2 |  |
| 3.13 Have you ever had sexual intercourse? | Yes 1  No 2 | If 2, skip to section to 4.1 |
| ***QUESTION 3.14 – 3.28 ARE ONLY FOR THOSE WHO HAVE EXPERIENCED PENETRATIVE SEX*** | | |
| - 1. Think back to the first time you had sexual intercourse. How would you describe your experience? | I forced my partner 1  I persuaded my partner 2  My partner persuaded me 3  My partner forced me 4  Both were willing 5 |  |
| 3.15 How old were you at the time you first had sex with your partner? | AGE (in years) |  |
| 3.16 Did you regret having intercourse with your partner on that first time? | Yes, regretted 1  No, not regretted 2 |  |
| 3.17 What did you use to avoid a pregnancy during your first sex? | Nothing 00 Condom 01  Pill 02  Injection 03  Withdrawal 04  Safe period 05  Other………………………….. 97 |  |
| 3.18 Did you ever discuss contraception with your partner? | YES, Before first intercourse 1  YES, After first intercourse 2  Never 3 |  |
| 3.19 How many times did you and partner have intercourse? (*estimate*) | Number:  Once only 1 | If 1, skip to 3.24 |
| 3.20 Apart from the first time, did you and your partner ever use a method to avoid pregnancy? | YES, Always 1  YES, Sometimes 2  Never 3 | If 3, skip to 3.24 |
| 3.21 What method did you and your partner mostly use? (MULTIPE RESPONSES PERMITTED) | Condom 1  Pill 2  Injection 3  Withdrawal 4  Safe period 5  Other…………………………….. 97 | If 4 or 5,  skip to 3.23 |
| 3.22 Where did you or your partner get this method? (CIRCLE ONLY ONE) | Shop 1  Pharmacy 2  Govt. Clinic/Health Centre/Hospital 3  Private Health worker/Nurse/Clinic 4  Friend 5  Other…………………………….. 97  Don't know 98 |  |
| - 1. Whose decision was it to use a method to prevent pregnancy? Was it mainly your decision, NAME'S decision or a joint decision? | My decision 1  My partner’s decision 2  Joint decision 3 |  |
| - 1. MALES: Did your partner ever become pregnant by you?   FEMALES: Did you ever become pregnant by your partner? | Yes 1  No 2 | if 2, skip to 3.26 |
| 3.25 What happened to the pregnancy? | Currently pregnant 1  Aborted 2  Miscarriage 3  Delivered 4  Not sure 5 |  |
| 3.26 Were you ever concerned that you might catch AIDS or another sexually transmitted disease from NAME? IF YES Very or somewhat? | Very concerned 1  Somewhat concerned 2  Not concerned 3 | If 3, skip to  SECTION 4 |
| 3.27 Were you able to do anything to reduce the risk of infection | Yes 1  No 2 | If 2, skip to SECTION 4 |
| 3.28 What did you do? *Probe* | Use condoms 01  Take medicines 02  Other (………………) 97 | Skip to SECTION 4 |

| **Section 4: Types of heterosexual contact** |
| --- |

| INTERVIEWER SEE Q. 3.1-3.3 ON PAGE 7  ANSWER TO 3.1 IS "NO": You told me that you have had no girl/boyfriends. I now want to ask you about any sexual contacts that you may have experienced.  ANSWER TO 3.1 IS "YES": You have told me about your relationship with NAME. Apart from her/him and any earlier girl /boyfriends, I now want to ask you about other types of sexual partners that you may have experienced. | |  |
| --- | --- | --- |
| 4.1 Some young people are forced to have sexual intercourse against their will by a stranger, a relative or an older person. Has this ever happened to you? | Yes 1  No 2 |  |
| 4.2 Some young people/females are touched on the breast or some other part of the body when they do not want to be, by a stranger, a relative or an older person. Has this ever happened to you? | Yes 1  No 2 | If 2, skip to 4.4 |
| 4.3 Would you say this has happened often, sometimes, or rarely? | Often 1  Sometimes 2  Rarely 3 |  |
| 4.4a Some young people pay money or gifts in exchange for sexual intercourse. Has this ever happened to you?  4.4b Some young people receive money or gifts in exchange for sexual intercourse. Has this ever happened to you? | Yes 1  No 2  Yes 1  No 2 | If 2 to 4.4a, skip to Section 5 **WHEN Q3.13 IS YES**  If 2 to 4.4b, skip to Section 5 **WHEN Q3.13 IS YES** |
| 4.5 How many women/men have you had sex with for money or gifts? | Number |  |

**THIS PAGE IS ONLY FOR THOSE WHO HAVE NEVER**

**EXPERIENCED SEXUAL INTERCOURSE**

| Ask these question only if answer to 3.13 is NO | | | | |
| --- | --- | --- | --- | --- |
| People may have mixed reasons for not having intercourse. I will read out some reasons. Please tell me for each reason whether it applies to you or not. | Applies | Not applies | Don't Know/ Not Sure |  |
| 4.6 I don't feel ready to have sex. | 1 | 2 | 3 |  |
| 4.7 I have not had the opportunity. | 1 | 2 | 3 |  |
| 4.8 I think that sex before marriage is wrong | 1 | 2 | 3 |  |
| 4.9 I am afraid of getting pregnant | 1 | 2 | 3 |  |
| 4.10 I am afraid of getting HIV/AIDS or another sexually transmitted infection. | 1 | 2 | 3 |  |
| - 1. Do you feel any pressure from others to have sexual intercourse? IF YES A great deal or a little? | A great deal 1  A little 2  None 3 | | | If 3, skip to Section 5 |
| - 1. From whom do you feel pressure? PROBE CIRCLE ALL THAT APPLY | Friends 01  Relatives 02  Work colleagues 03  Partner/special friend 04  Other ………………………………………… 97. | | |  |
|  |  | | |  |

| **Section 5: Knowledge and ever-use of contraceptive methods** |
| --- |

| I now have some questions about contraception - I mean ways in which men and women can avoid getting pregnant. Which methods have you heard of? What others?  CIRCLE CODE 1 IN COL. 2 FOR EACH METHOD MENTIONED.  FOR EACH METHOD IN THE TABLE NOT ALREADY MENTIONED, READ THE DESCRIPTION IN COL.1 AND RECORD ANSWER IN COL.2  FOR EACH METHOD KNOWN ASK QUESTION IN COL.3 | | | | |
| --- | --- | --- | --- | --- |
| **COL 1.** | **COL. 2.** | | **COL. 3.** | |
| - 1. Pill   Women can take a pill every day | Knowledge of Method  Yes (spont.) 1  Yes (prompted) 2  No 3 | | Knowledge of Source  "Do you know any place or person where young people could obtain this method?  Yes 1  No 2 | |
| 5.2 Injection  Women can have an injection every 2 or every 3 months | Yes (spont.) 1  Yes (prompted) 2  No 3 | | "Do you know any place or person where young people could obtain this method?  Yes 1  No 2 | |
| 5.3 Condom  A man can put a rubber device on his penis before intercourse | Yes (spont.) 1  Yes (prompted) 2  No 3 | | "Do you know any place or person where young people could obtain this method?  Yes 1  No 2 | |
| 5.4 Emergency Contraceptive Pills  A woman can take pills soon after intercourse | Yes (spont.) 1  Yes (prompted) 2  No 3 | | "Do you know any place or person where young people could obtain this method?  Yes 1  No 2 | |
| 5.5 Withdrawal  A man can pull out of a woman before climax | Yes (spont.) 1  Yes (prompted) 2  No 3 | |  | |
| 5.6 There are other methods of contraception that I have not mentioned. What other methods have you heard of? CIRCLE EACH METHOD MENTIONED. | | IUD 01  Implant 02  Jelly/foam 03  Female Sterilization 04  Male Sterilization 05  Periodic Abstinence 06  Other (SPECIFY)……………….. 97  Don’t know anymore 98 | |  |
| - 1. Which method do you think is most suitable for young people?   CIRCLE ONE ANSWER | | Pill 01  injection 02  Condom 03  Emergency. Pills 04  Withdrawal 05  Periodic abstinence. 06  Other …………………………….. 07  Don’t Know 98 | |  |
| - 1. SEE Q3.13   Respondent has Respondent has not  experienced sexual experienced sexual  intercourse intercourse | | | | SECTION 6 |
| - 1. Which methods of contraception have you or a sexual partner ever used?   PROBE which others?  CIRCLE ALL THAT APPLY | | Pill 01  injection 02  Condom 03  Emergency Pills 04  Withdrawal 05  Periodic abstinence. 06  Other ……………………… 97 | |  |

| **Section 6: Knowledge of HIV/AIDS and sexually transmitted diseases** |
| --- |

| 6.1 Have you heard of HIV or AIDS (*use local terms*)? | Yes 1  No 2 | | | | If 2, skip to 6.5 |
| --- | --- | --- | --- | --- | --- |
| I am now going to read you some statements about HIV/AIDS. Please tell me whether you think the statement is true, or false, or whether you don't know. | True | | False | Don't know |  |
| 6.2 It is possible to cure AIDS | 1 | 2 | | 3 |  |
| 6.3 A person with HIV always looks emanciated or unhealthy in some way | 1 | 2 | | 3 |  |
| 6.4 People can take a simple test to find out whether they have HIV | 1 | 2 | | 3 |  |
| 6.5 Apart from HIV/AIDS, there are other diseases that men and women can catch by having sexual intercourse. Have you heard of any of these diseases? | Yes 1  No 2 | | | | If 2, skip to SECTION 7 |
| - 1. What are the signs and symptoms of a sexually transmitted disease in a man? PROBE   CIRCLE EACH MENTIONED | Discharge from penis 01  Pain during urination 02  Ulcers/sores in genital area 03  Other…………………… 97  Don’t Know any signs 98 | | | |  |
| 6.7 And what are the signs or symptoms when a woman is infected? | Vaginal discharge 01  Pain during urination 02  Ulcers/sores in genital area 03  Other…………………… 97  Don’t Know any signs 98 | | | |  |
| 6.8 If a friend of yours needed treatment for a sexually transmitted disease, where could he or she obtain such treatment? PROBE Any other places?  CIRCLE EACH MENTIONED | Shop 01  Pharmacy 02  Govt. hospital/health center 03  Private health worker/nurse/ 04  Other (specify)…………… 97 | | | |  |

| **Section 7: Condom knowledge and attitudes** |
| --- |

| - 1. SEE Q.5.3 ON PAGE 12   CONDOMS KNOWN | CONDOMS NOT KNOWN | | | SECTION 8 |
| --- | --- | --- | --- | --- |
| - 1. SEE Q3.13   Respondent has experienced sexual intercourse | Respondent has not  experienced sexual  intercourse | | | 7.5 |
| - 1. Have you or a partner ever used a condom? | Yes 1  No 2 | | | If 2, skip to 7.6 |
| - 1. Have you ever experienced a condom that split or broke during intercourse? | Yes 1  No 2 | | | Move to 7.6 |
| - 1. Have you ever seen a condom? | Yes 1  No 2 | | |  |
| People have different opinions about condoms. I will read out some opinions. For each one, I want you to tell me whether you agree or disagree, or whether you don't know | Agree | Don't know/not sure | Disagree |  |
| - 1. Condoms are an effective method of preventing pregnancy | 1 | 2 | 3 |  |
| - 1. A condom can be used more than once | 1 | 2 | 3 |  |
| - 1. A girl can suggest to her boyfriend that he uses a condom | 1 | 2 | 3 |  |
| - 1. A boy can suggest to his girlfriend that he uses a condom | 1 | 2 | 3 |  |
| - 1. Condoms are an effective way of protecting against HIV/AIDS | 1 | 2 | 3 |  |
| - 1. It would be too embarrassing for someone like me to buy or obtain condoms | 1 | 2 | 3 |  |
| - 1. If a girl suggested using condoms to her partner, it would mean that she didn't trust him | 1 | 2 | 3 |  |
| - 1. Condoms reduce sexual pleasure | 1 | 2 | 3 |  |
| - 1. If unmarried couples want to have sexual intercourse before marriage, they should use condoms | 1 | 2 | 3 |  |
| - 1. Condoms are an effective way of protecting against sexually transmitted diseases | 1 | 2 | 3 |  |

| **Section 8: Sexuality, gender and norms** |
| --- |

| Young people have various views about relationships. I will read out to you some views. For each one, please tell me whether you agree or disagree? |  |  |
| --- | --- | --- |
| 8.1 I believe it's all right for unmarried boys and girls to have dates (USE LOCAL TERM) | Agree 1  Don’t know/not sure 2  Disagree 3 |  |
| 8.2 I believe it's all right for boys and girls to kiss, hug and touch each other. | Agree 1  Don’t know/not sure 2  Disagree 3 |  |
| 8.3 I believe there is nothing wrong with unmarried boys and girls having sexual intercourse if they love each other. | Agree 1  Don’t know/not sure 2  Disagree 3 |  |
| 8.4 I think that sometimes a boy has to force a girl to have sex if he loves her. | Agree 1  Don’t know/not sure 2  Disagree 3 |  |
| 8.5 A boy will not respect a girl who agrees to have sex with him. | Agree 1  Don’t know/not sure 2  Disagree 3 |  |
| 8.6 Most girls who have sex before marriage regret it afterwards. | Agree 1  Don’t know/not sure 2  Disagree 3 |  |
| 8.7 Most boys who have sex before marriage regret it afterwards. | Agree 1  Don’t know/not sure 2  Disagree 3 |  |
| 8.8 A boy and a girl should have sex before they become engaged (USE LOCAL TERM) to see whether they are suited to each other. | Agree 1  Don’t know/not sure 2  Disagree 3 |  |
| 8.9 I believe that girls should remain virgins until they marry. | Agree 1  Don’t know/not sure 2  Disagree 3 |  |
| 8.10 I believe that boys should remain virgins until they marry. | Agree 1  Don’t know/not sure 2  Disagree 3 |  |
| 8.11 It's all right for boys and girls to have sex with each other provided that they use methods to stop pregnancy. | Agree 1  Don’t know/not sure 2  Disagree 3 |  |
| 8.12 Most of my friends who have sex with someone use condoms regularly. | Agree 1  Don’t know/not sure 2  Disagree 3 |  |
| 8.13 I am confident that I can insist on condom use every time I have sex. | Agree 1  Don’t know/not sure 2  Disagree 3 |  |
| 8.14 I would never contemplate having an abortion my self or for my partner. | Agree 1  Don’t know/not sure 2  Disagree 3 |  |
| 8.15 It is mainly the woman's responsibility to ensure that contraception is used regularly. | Agree 1  Don’t know/not sure 2  Disagree 3 |  |
| 8.16 I think that you should be in love with someone before having sex with them. | Agree 1  Don’t know/not sure 2  Disagree 3 |  |
| 8.17 I feel that I know how to use a condom properly. | Agree 1  Don’t know/not sure 2  Disagree 3 |  |
| 8.18 Most of my friends would never contemplate having an abortion for themselves or their partner. | Agree 1  Don’t know/not sure 2  Disagree 3 |  |
| 8.19 Most of my friends believe that you should be in love before you have sex with someone. | Agree 1  Don’t know/not sure 2  Disagree 3 |  |
| 8.20 I would refuse to have sex with someone who is not prepared to use a condom. | Agree 1  Don’t know/not sure 2  Disagree 3 |  |
| 8.22 How many of your friends have had sexual intercourse? Would you say many, some, a few, or none? | Many 1  Some 2  A few 3  None 4  Not sure 8 |  |

| **Section 9: Use and perceptions of health services** |
| --- |

| - 1. Have you ever visited a health facility or health worker of any kind to receive services or information on contraception, pregnancy, abortion or sexually transmitted dieases? | Yes 1  No 2 | | If 2, move to END |
| --- | --- | --- | --- |
| - 1. How many times have you sought services or information from a health worker or a nurse for these services in the last twelve months? | Number of times  Did not seek care in last 12 months 0 | | If 0, move to END |
| - 1. Thinking about your last visit, did you go to a government clinic, health centre or hospital or a private health worker or clinic? | Government 1  Private 2  Other…………………………………. 3 | |  |
| - 1. When you last saw a health worker or a nurse, what was your reason for going? | Contraception 1  STD 2  Gynecological exam 3  Pregnancy test 4  Pregnancy termination 5  Maternal and Child Health 6  Other……………………………… 7 | |  |
| - 1. At this facility   Did you see any posters on contraception? | YES NO NOT SURE  1 2 3 | |  |
| - 1. Did the health worker or nurse talk to you about:  1. Contraception? 2. Sexually transmitted diseases? 3. Pregnancy? | YES NO NOT SURE  1 2 3  1 2 3  1 2 3 | |  |
| - 1. Did you feel comfortable enough to ask questions? | 1 | 2 | If 2, move to 9.10 |
| - 1. Were the questions you asked during the consultation answered adequately? | 1 | 2 |  |
| - 1. Was there enough confidentiality? | 1 | 2 |  |

**END TIME FOR INTERVIEW HOURS MINS**

**Do you have any question or comments for me?**

....................................................................................................................................................................................................................................................................................................................................................................................................................................................................................

**WE HAVE COME TO THE END OF THE INTERVIEW, THANK YOU VERY MUCH FOR TAKING YOUR TIME TO ANSWER THESE QUESTIONS**

**END**
